# Optimizing endoscopic detection of precancerous gastric conditions: Single-center prospective study

**DOI:** 10.1055/a-2557-6356

**Published:** 2025-04-04

**Authors:** Jennifer Aoun, Elena Unger, Mohamed Abdessalami, Amélie Bourgeois, Maria Galdon Gomez, Laurine Verset, Mariana Figueiredo, Pierre Eisendrath

**Affiliations:** 1 81880 Department of Gastroenterology, Hepatopancreatology and Digestive Oncology, CHU Saint-Pierre, Brussels, Belgium; 2 60210 Department of Pathology, Institut Jules Bordet, Brussels, Belgium

**Keywords:** Endoscopy Upper GI Tract, Precancerous conditions & cancerous lesions (displasia and cancer) stomach, Diagnosis and imaging (inc chromoendoscopy, NBI, iSCAN, FICE, CLE)

## Abstract

**Background and study aims:**

Chronic atrophic gastritis is an asymptomatic precancerous condition that can progress to extensive atrophy and/or intestinal metaplasia (IM), referred to as advanced stage of atrophic gastritis (ASAG). ASAG is a common condition with a variable prevalence worldwide reaching 45%. Narrow-band imaging (NBI) already has an established role in improving endoscopic detection of atrophy and IM. Considering the heterogeneous hospital population, this study aimed to assess the ASAG detection rate with NBI-guided biopsies compared with conventional Sydney protocol, in a European cosmopolitan city hospital.

**Patients and methods:**

This was a prospective, single-center, bi-phasic study conducted between October 2023 and March 2024, comparing ASAG detection rates using conventional Sydney protocol with optional NBI use, defined as phase 1, versus systematic NBI-guided biopsies in phase 2.

**Results:**

Of 495 eligible patients, 435 with similar demographics were included in both phases (87.8%). ASAG was detected in three patients using conventional Sydney protocol (1.43%) compared with eight patients (3.56%) using systematic NBI-guided biopsies ( *P* = 0.269). Furthermore, systematic NBI-guided biopsies were associated with increased detection rates for atrophy and IM ( *P* = 0.223 and *P* = 0.502, respectively). Suspicion-free NBI use correlated with increased likelihood of ASAG detection (odds ratio 16.99, 95% confidence interval 2.30–213.73). Age ≥ 50 years was a significant risk factor associated with ASAG.

**Conclusions:**

Despite the diverse hospital population, ASAG prevalence remained low. A numerical increase in ASAG detection rate was observed with systematic NBI use compared with optional NBI use. Overall, systematic NBI-guided biopsies appear to be associated with increased rates of detection of ASAG, atrophy, and IM.

## Introduction

Gastric cancer (GC) continues to be a significant health concern, ranking fifth for cancer-related mortality worldwide and in Europe 1 . Absent screening recommendations for the general population in Western countries, proactive identification and subsequent endoscopic follow-up of high-risk individuals is warranted, mainly those with advanced stages of atrophic gastritis (ASAG) 2 .

ASAG is a precancerous condition, representing a progressive phase of the Correa cascade that can ultimately progress to gastric adenocarcinoma. It is defined as extensive involvement of atrophy and/or intestinal metaplasia (IM), affecting both the antral and corpus mucosa 2 . ASAG is a common condition with a varying prevalence in the literature between 7% and 45% 3 4 . The most frequent risk factors are *Helicobacter pylori* chronic infection and, to a lesser extent, autoimmune gastritis. Other risk factors for ASAG include age, tobacco use, high salt diet, and possibly chronic biliary reflux 5 .

Following upper gastrointestinal endoscopy (UGIE) with gastric biopsies according to the Sydney protocol, Operative Link on Gastritis Assessment (OLGA) and Operative Link on Gastric Intestinal Metaplasia (OLGIM) histologic scores allow classification of atrophy and metaplasia respectively, based on degree of severity. For the purpose of this study, ASAG will be defined as OLGA/OLGIM III 2 .

Although white light endoscopy (WLE) provides good visibility of focal gastric lesions, it may not be optimal for detection of IM, which appears as gray-white nodular changes in gastric mucosa 6 7 . Virtual chromoendoscopy, in contrast, highlights surface microvasculature, revealing light blue crest appearances with narrow band imaging (NBI), which is considered highly specific for IM 8 9 .

Some studies suggest higher detection rates for IM using NBI-guided targeted biopsies 4 6 10 . However, the advantage of systematic use of NBI-guided biopsies over the conventional Sydney protocol has only been demonstrated in studies with small samples. Moreover, to our knowledge, no prospective comparative study has been conducted in Belgium to date. Being a Belgian public hospital at the center of Brussels, Saint-Pierre University Hospital attracts a diverse population from various geographic origins compared with nearby hospitals, including many patients arriving from regions of high ASAG prevalence.

The aim of our study was to evaluate the impact of implementing NBI-guided biopsies on the detection rate for ASAG in a cosmopolitan city university hospital in Brussels, Belgium.

## Patients and methods

### Study aims

This study hypothesized that systematic use of NBI-enhanced gastric tissue sampling improves detection of ASAG, thereby improving identification of precancerous gastric conditions. Hence, the primary outcome was the difference in detection rate for ASAG using systematic NBI-guided gastric biopsies compared with the conventional Sydney protocol.

Secondary objectives were: 1) evaluation of the difference in detection rates for atrophy and IM using NBI-guided gastric biopsies compared with the conventional Sydney protocol; 2) prospective evaluation of prevalence of ASAG, atrophy, and intestinal metaplasia in our population; 3) assessment of risk factors associated with ASAG, atrophy and IM; and 4) *H. pylori* infection prevalence among the diverse hospital population.

### Study design

This was a single-center prospective study conducted at Saint-Pierre University Hospital between October 2023 and March 2024. Eight gastroenterologists with different levels of endoscopic experience were invited to participate. Patients older than 18 years of age undergoing UGIE were considered eligible for the study and were given a written informed consent to sign before endoscopic examination. Indications for UGIE mostly included dyspepsia, abdominal pain, reflux, anemia, and pre-bariatric surgery. Patients with a prior history of gastric surgery, known severe gastritis currently in a follow-up program, or a previous history of GC were excluded. Hospitalized patients and those who underwent UGIE under general anesthesia were also excluded. Pregnancy was not an exclusion criterion.

Pre-procedure administration of simethicone was mandatory according to study protocol and department standard practice 11 . Sedation with small doses of intravenous midazolam was administered at endoscopist discretion. Procedures were conducted using high-definition Olympus endoscopes (H/HQ190 series), all equipped with NBI functionality.

At the end of the procedure, endoscopists were requested to complete a short electronic questionnaire on REDCap. The recorded data included patient demographics, procedure description, and pathology results. Data regarding family history of GC were collected based on patient interviews.

The study was divided into two distinct phases. The first phase started in October 2023 with an informative presentation to the participating endoscopists, focused on ASAG prevalence, risk factors, and complications. During this phase, UGIE biopsies were performed according to standard practice using the conventional Sydney protocol. Use of NBI-guided biopsies was intentionally left unmentioned at this stage in order to guarantee an unbiased view of the baseline situation (observatory bias). During this phase, we evaluated baseline prevalence of ASAG as well as the rate of optional NBI use. The endoscopists were instructed to report any suspicion of gastric atrophy and/or IM under WLE preceding NBI use.

**Fig. 1:**
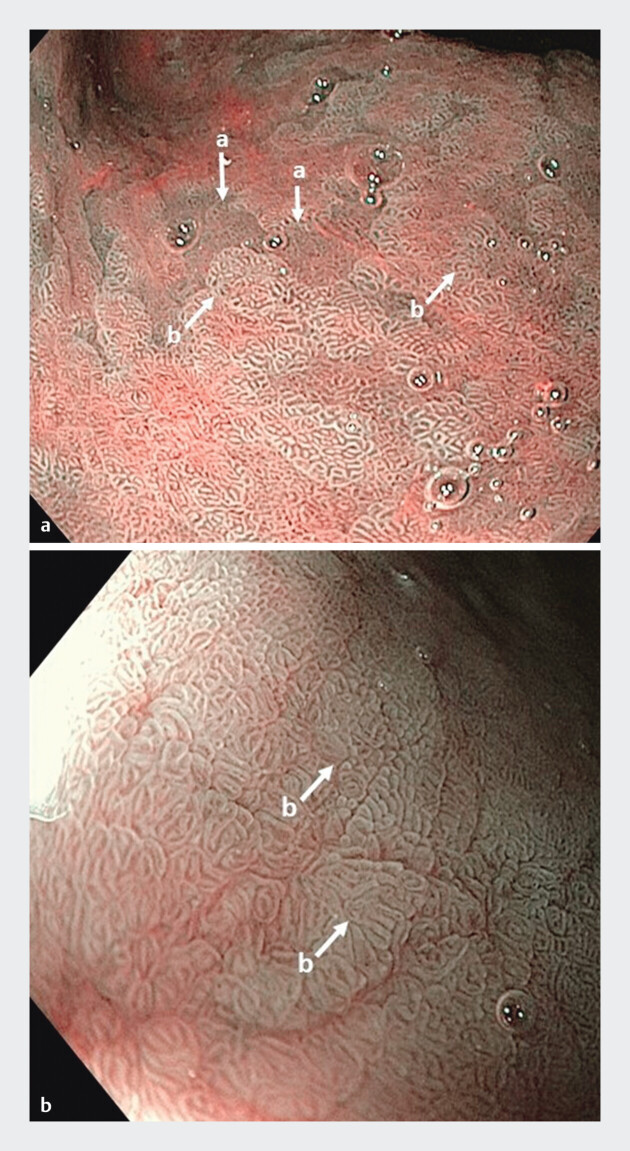
Endoscopic patterns of gastric atrophy ( **a** ) and intestinal metaplasia ( **b** ) under narrow band imaging (NBI).

The first phase ended on December 18, 2023, with an instructive presentation provided by an endoscopist with expertise in chromoendoscopy, not a recruiter in the study. The presentation featured theoretical information along with visual aids designed to improve detection of atrophy and IM using NBI ( Fig. 1 ). Presence of dilated coiled subepithelial capillaries, regular ridged surface structures, and loss of gastric pits indicated presence of gastric atrophy, whereas regular tubulovillous mucosal pattern or light blue crest sign were suggestive of IM 12 .

The second phase of the study began on December 19, 2023 and ended on March 29, 2024. During this phase, endoscopists were instructed to consistently apply NBI function in all UGIEs, and to perform NBI-guided biopsies. In an effort to reinforce endoscopist training, we developed image recognition quizzes facilitating identification of atrophy and mostly IM with NBI.

### Histopathological evaluation

Two senior gastrointestinal pathologists blinded to endoscopic examination details reviewed the gastric biopsies specimens. Evaluation for presence of *H. pylori* was performed using immunohistochemical staining. OLGA and OLGIM grading systems were included in all pathological reports.

### Ethics concerns

The institution ethics committee (B0762023230705) approved the study protocol. The study did not impose any additional risks to patients care. Nevertheless, participants were notified that the endoscopic examination might be slightly extended, particularly in phase 2, if the endoscopist had not yet fully mastered the protocol.

### Statistical analysis

For the purpose of sample size calculation, we referred to prevalence of ASAG in the population of patients at Saint-Pierre University Hospital, estimated to be around 2% based on retrospective data. Based on a recent multicenter prospective study evaluating prevalence of ASAG in a European population 4 , we postulated a projection for improvement of detection to 8% during phase 2 of the study. According to this detection variation, we planned to include 206 patients in each phase. This number was calculated to allow a statistical power of 80% with a 95% confidence interval (CI).

Continuous variables were expressed as mean (standard deviation) whereas categorical variables were expressed as counts (percentages). Demographic and clinical characteristics were compared between groups using Fisher’s exact test or the chi-squared test for categorical variables and the Student’s *t* test for continuous variables. Odds ratios (ORs) along with their 95% CIs were estimated using binary logistic regression. All tests were two-sided, and statistical significance was set at 5% level. REDCap and Rstudio (R software, version 4.2.2) were used for data support and analysis.

### Potential bias

Because patients did not serve as their own controls, election bias between phase one and phase two cannot be excluded.

## Results

**Fig. 2:**
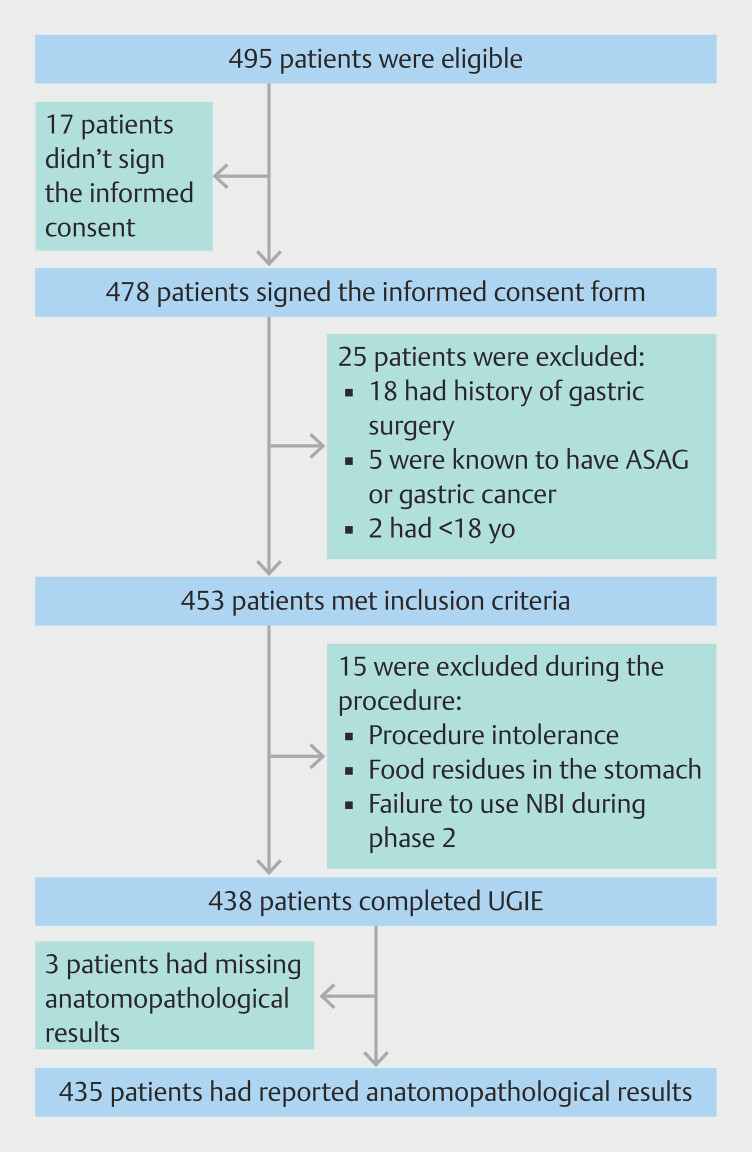
Flowchart of patient selection.

Of the 495 eligible patients, 435 (87.8%) were included in both phases. Sixty patients were excluded for different reasons, summarized in Fig. 2 .

### Patient demographics

A total of 210 patients were included in phase 1 and 225 in phase 2. Patient characteristics are shown in Table 1 .

**Table:** **Table 1** Characteristics of included patients.

	Overall population N = 435	Phase 1 N = 210	Phase 2 N = 225	Overall *P* value
Age, years (SD)	46.6 (15.9)	46.5 (15.8)	46.7 (15.9)	0.921
Age ≥ 50 years (%)	172 (39.5)	82 (39)	90 (40.0)	0.916
Gender male (%)	188 (43.2)	82 (39)	106 (47.1)	0.110
Geographical origin				
North Europe (%)	163 (37.5)	73 (34.8)	90 (40.0)	
North Africa (%)	109 (25.1)	57 (27.1)	52 (23.1)	
South Europe (%)	32 (7.4)	17 (8.1)	15 (6.7)	
Other (%)	131 (30.1)	63 (30.0)	68 (30.2)	
BMI				0.359
Underweight BMI < 18 (%)	5 (1.15)	4 (1.9)	1 (0.44)	
Normal weight BMI 18–25 (%)	268 (61.6)	124 (59)	144 (64.0)	
Overweight BMI 25–30 (%)	105 (24.1)	51 (24.3)	54 (24.0)	
Obese BMI > 30 (%)	57 (13.1)	31 (14.8)	26 (11.6)	
Smoking (%)	107 (24.6)	57 (27.1)	50 (22.2)	0.137
Alcohol consumption (%)	43 (9.9)	23 (11.0)	20 (8.9)	0.470
Family history of gastric cancer (%)	19 (4.4)	9 (4.3)	10 (4.4)	0.436
BMI, body mass index; SD, standard deviation.				

The two populations were similar in terms of demographics. Mean age in phase 1 was 46.5 compared with 46.7 in phase 2 ( *P* = 0.921). There were fewer male patients in phase 1 (39%), but the sex ratio was balanced in phase 2, where 47% of patients were male. However, this difference was not statistically significant ( *P* = 0.11). In both phases, the geographic origin of patients was predominantly North European.

Most patients in phase 1 (59%) and in phase 2 (64%) had a normal body mass index (BMI) ranging between 18 and 25. The proportion of patients with a BMI > 30 was similar in both phases (14.8% and 11.6% respectively, *P* = 0.359). A first-degree family history of GC was reported in 4.29% of patients in phase 1 compared with 4.44% in phase two ( *P* = 0.436).

### Procedure

Endoscopists with less than 10 years of experience (N = 3) performed 47.6% of UGIE in phase 1 compared with 48.9% in the second part ( *P* = 0.866).

Using WLE, operators suspected gastric atrophy in 15.7% and 21.3% in phase 1 and 2, respectively ( *P* = 0.167). The location of the suspected atrophy was similar in both phases, with a clear predominance of the isolated antrum location (69.7% vs 77.1%).

WLE resulted in suspicion for IM in fewer than 10% of patients in both phases, namely 16 cases (7.62%) in phase 1 compared with 20 cases (8.89%) in the phase 2 ( *P* = 0.759). Suspected IM displayed a location pattern similar to that for atrophy. NBI was applied in 62 UGIEs during phase 1 (29.5%) and in all UGIEs during phase 2 (100%) ( Table 2 ).

**Table:** **Table 2** Procedure characteristics and pathology results for phase 1 and phase 2.

	Overall population N = 435	Phase 1 N = 210	Phase 2 N = 225	Overall *P* value
Endoscopist experience < 10 years (%)	210 (48.3)	100 (47.6)	110 (48.9)	0.866
Sedation				
Local spray (%)	283 (65.1)	141 (67.1)	142 (63.1)	0.435
IV midazolam (%)	152 (34.9)	69 (32.9)	83 (36.9)	
Suspected IM WLE (%)	36 (8.28)	16 (7.62)	20 (8.9)	0.759
Suspected atrophy WLE (%)	81 (18.6)	33 (15.7)	48 (21.3)	0.167
NBI use (%)	287 (66.0)	62 (29.5)	225 (100)	**< 0.001**
Suspected IM NBI (%)	37 (12.9)	2 (3.2)	35 (15.5)	**0.010**
Suspected atrophy NBI (%)	21 (7.3)	5 (8)	16 (7.1)	0.798
H. pylori infection (%)	131 (30.1)	66 (31.4)	65 (28.9)	0.637
Autoimmune gastritis (%)	0	0	0	
Chronic gastritis (%)	342 (78.6)	171 (81.4)	171 (76)	0.207
OLGA				0.130
OLGA 0 (%)	269 (78.7)	141 (82.5)	128 (74.9)	
OLGA 1/2 (%)	62 (18.1)	27 (15.7)	35 (20.4)	
OLGA 3/4 (%)	11 (3.2)	3 (1.75)	8 (4.6)	
OLGIM				0.090
OLGIM 0 (%)	265 (77.5)	137 (80.1)	128 (74.8)	
OLGIM 1/2 (%)	70 (14)	33 (19.2)	37 (21.6)	
OLGIM 3/4 (%)	7 (1.75)	1 (0.58)	6 (3.5)	
Primary endpoint				0.269
ASAG (%)	11 (2.53)	3 (1.43)	8 (3.56)	
ASAG, advanced stage of atrophic gastritis; IM, intestinal metaplasia; IV, intravenous; NBI, narrow-band imaging; OLGA, Operative Link on Gastritis Assessment; OLGIM, Operative Link on Gastric Intestinal Metaplasia; WLE, white light endoscopy.				

### Pathology

In the studied population, 78.6% presented with chronic gastritis, 16.8% with atrophy, 17.7% with IM, and 2.53% with ASAG ( Table 2 ). ASAG (OLGA/OLGIM ≥ III) was found in three patients in phase 1 (1.43%) compared with eight patients in phase 2 (3.56%) ( *P* = 0.269). *H. pylori* was detected by immunostaining in 31.4% of patients in phase 1 compared with 28.9% in the phase 2 ( *P* = 0.637).

**Fig. 3:**
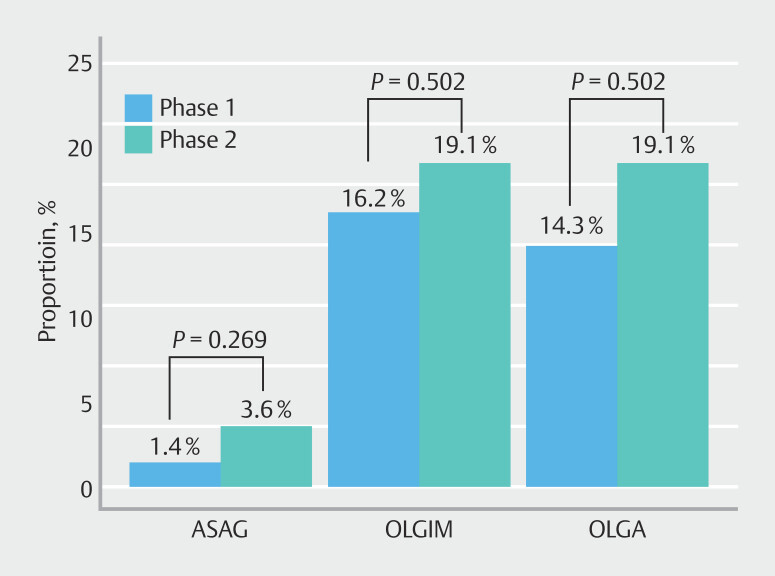
Comparison of ASAG, OLGIM-positive, and OLGA-positive between the two phases.

In addition, a numerical increase in identification of atrophy and IM (all stages combined) was noted between phase 1 and phase 2 ( Fig. 3 ).

During phase 1, optional NBI use allowed endoscopists to suspect a single case of atrophy and none of IM, previously unsuspected in WLE. This compares with nine cases of newly suspected atrophy and 29 cases of newly suspected IM with the systematic use of NBI during phase 2. After matching these results with their corresponding pathology results, this translated into an increase in sensitivity for detection of both pathologies, but with statistical significance only for IM ( *P* < 0.001). Specificity for detection of both pathologies decreased in phase 2, statistically significant for IM ( *P* = 0.015) ( Table 3 ).

**Table:** **Table 3** Comparison of sensitivity and specificity for detection of atrophy and IM between phases.

		**Atrophy**			**IM**	
	Phase 1	Phase 2	*P* value	Phase 1	Phase 2	*P* value
Sensitivity	36.7%	51.2%	0.221	23.5%	65.1%	< 0.001
Specificity	87.2%	80.8%	0.094	95.5%	88.5%	0.015
IM, intestinal metaplasia.						

We performed a univariate analysis for associated factors with ASAG detection, as shown in Table 4 . Ten cases of ASAG were detected in the population of patients older than age 50 years versus one case in the population younger than age 50 years. This implies a significant elevation in risk of ASAG with age 50 (odds ratio [OR] 8.04, 95% CI 2.02–63.3; *P* = 0.001).

**Table:** **Table 4** Univariate analysis of risk factors associated with ASAG.

	No ASAG N = 424	ASAG N = 11	OR (95% CI)	Overall *P* value
Age, years (SD)	46.3 (15.9)	57.5 (12)	1.04 (1.01;1.08)	**0.011**
Age ≥ 50 years (%)	162 (38.2)	10 (90.9)	8.04 (2.02;63.3)	**0.001**
Gender Male (%)	183 (43.2)	5 (45.5)	0.94 (0.35;3.52)	1.000
Geographical origin				0.446
High-risk countries (%)	77 (18.2)	3 (27.3)	1.69 (0.43;6.51)	
BMI				0.271
Overweight (%)	104 (24.5)	1 (9.1)	0.22 (0.06;1.98)	
Obese (%)	57 (13.4)	0 (0)	0.00 (0.01;3.71)	
Smoking (%)	103 (24.3)	4 (36.4)	1.53 (0.56;6.09)	0.503
Alcohol (%)	41 (9.67)	2 (18.2)	1.82 (0.58;10.1)	0.316
Family history of gastric cancer (%)	18 (4.25)	1 (9.1)	1.92 (0.53;18.3)	0.468
Endoscopist experience > 10 years (%)	222 (52.4)	3 (27.3)	0.30 (0.11;1.32)	0.181
Systematic NBI-guided biopsies (%)	217 (51.2)	8 (72.7)	1.90 (0.66;8.16)	0.269
*H. pylori* infection (%)	128 (30.2)	3 (27.3)	0.76 (0.27;3.36)	1.000
ASAG, advanced stage atrophic gastritis; BMI, body mass index; CI, confidence interval; NBI, narrow-band imaging; OR, odds ratio; SD, standard deviation.				

In our study population, high-risk geographical origins included Asia, Middle East, South Europe, and South America 1 . Although high-risk origins were associated with an increased risk of ASAG, the results were not statistically significant (OR 1.69, 95% CI 0.43–6.51; *P* = 0.446). The systematic NBI-guided biopsy protocol implemented in phase 1 was associated with increased odds of ASAG detection compared with the conventional Sydney protocol applied in phase 1 (OR 1.90, 95% CI 0.66–8.16; *P* = 0.269). *H. pylori* status was not shown to increase risk of ASAG (OR 0.76, 95% CI 0.27–3.36; *P* = 1.000) ( Table 4 ).

We conducted a subgroup analysis for patients in whom use of NBI was unaffected by prior suspicion of atrophy and/or IM with WLE. This subgroup, referred to as “suspicion-free” NBI use, combined unbiased NBI use from phase 1 and all patients with systematic NBI use from phase 2. Patients from phase 1, where NBI use was determined by preceding suspicion of atrophy and/or IM under WLE, were excluded. It was found that “suspicion-free” NBI use was significantly associated with increased likelihood of ASAG detection (OR 16.99, 95% CI 2.30–213.73; *P* = 0.016) ( Table 5 ).

**Table:** **Table 5** Association between “suspicion-free” NBI use and ASAG detection rate.

	No ASAG N = 406	ASAG N = 8	OR (95% CI)	Overall *P* value
Suspicion-free NBI use (%)	258 (63.5)	8 (100)	16.99 (2.30–213.73)	**0.016**
ASAG, advanced stage of atrophic gastritis; CI, confidence interval; NBI, narrow-band imaging; OR, odds ratio.				

We performed the same analysis for associated factors with presence of atrophy (OLGA > 0) and IM (OLGIM > 0) ( Table 6 and Table 7 ).

**Table:** **Table 6** Univariate analysis of risk factors associated with atrophy (all stages combined).

	No atrophy N = 362	Atrophy (OLGA > 0) N = 73	OR (95% CI)	Overall *P* value
Age, years (SD)	44.8 (15.3)	55.7 (15.6)	1.04 (1.03–1.06)	**< 0.001**
Age ≥ 50 years (%)	125 (34.5)	47 (64.4)	3.27 (2.01–5.72)	**< 0.001**
Gender male (%)	151 (41.7)	37 (50.7)	1.39 (0.87–2.37)	0.200
Geographical origin				
High-risk countries (%)	62 (17.1)	18 (24.7)	1.58 (0.87–2.88)	0.132
BMI				0.718
Overweight (%)	88 (24.3)	17 (23.3)	0.86 (0.49–1.64)	
Obese (%)	50 (13.8)	7 (9.59)	0.62 (0.30–1.54)	
Smoking (%)	91 (25.1)	16 (21.9)	0.82 (0.48–1.57)	0.336
Alcohol (%)	29 (8.01)	14 (19.2)	2.58 (1.38–5.46)	**0.012**
Family history of gastric cancer (%)	17 (4.70)	2 (2.74)	0.52 (0.18–2.62)	0.715
Endoscopist experience > 10 years (%)	194 (53.6)	31 (42.5)	0.62 (0.39–1.06)	0.108
Systematic NBI-guided biopsies (%)	182 (50.3)	43 (58.9)	1.36 (0.85–2.34)	0.223
*H. pylori* infection (%)	108 (29.8)	23 (31.5)	1.05 (0.64–1.87)	0.885
BMI, body mass index; CI, confidence interval; NBI, narrow-band imaging; OLGA, Operative Link on Gastritis Assessment; OR, odds ratio; SD, standard deviation.				

**Table:** **Table 7** Univariate analysis of risk factors associated with IM (all stages combined).

	No IM N = 358	IM (OLGIM > 0) N = 73	OR (95% CI)	Overall *P* value
Age, years (SD)	44.5 (15.2)	56.3 (15.4)	1.05 (1.03;1.07)	**< 0.001**
Age ≥ 50 years (%)	121 (33.8)	51 (66.2)	3.67 (2.27;6.37)	**< 0.001**
Gender male (%)	151 (42.2)	37 (48.1)	1.23 (0.78;2.07)	0.349
Geographical origin				
High-risk countries (%)	61 (17.0)	19 (24.7)	1.59 (0.88;2.86)	0.119
BMI				0.642
Overweight (%)	87 (24.3)	18 (23.4)	0.85 (0.50;1.60)	
Obese (%)	50 (13.8)	7 (9.09)	0.57 (0.27;1.43)	
Smoking (%)	90 (25.1)	17 (22.1)	0.83 (0.48;1.56)	0.352
Alcohol (%)	29 (8.10)	14 (18.2)	2.39 (1.28;5.04)	**0.020**
Family history of gastric cancer (%)	17 (4.70)	2 (2.74)	0.52 (0.18;2.62)	0.565
Endoscopist experience > 10 years (%)	190 (53.1)	35 (45.5)	0.72 (0.45;1.21)	0.277
Systematic NBI-guided biopsies (%)	182 (50.8)	43 (55.8)	1.18 (0.75;2.00)	0.502
*H. pylori* infection (%)	108 (30.2)	23 (29.9)	0.96 (0.58;1.70)	1.000
BMI, body mass index; CI, confidence interval; IM, intestinal metaplasia; NBI, narrow-band imaging; SD, standard deviation.				

Age ≥ 50 years was a risk factor for atrophy and IM (OR 3.27, 95% CI 2.01–5.72; *P* < 0.001) and (OR 3.67, 95% CI 2.27–6.37; *P* < 0.001), respectively. Excessive alcohol consumption (defined as > 8 units/week for women and > 15 units/week for men) 13 increased risk of atrophy (OR 2.58, 95% CI 1.38–5.46; *P* = 0.012) and IM (OR 2.39, 95% CI 1.28–5.04; *P* = 0.020).

## Discussion

The main findings from this single-center prospective study are the following: 1) prevalence of ASAG was lower than estimated in our population of patients; and 2) systemic use of NBI-guided biopsies was associated with a numerical increase in detection rate for ASAG compared with the conventional Sydney protocol with optional NBI use. Nevertheless, “suspicion-free” NBI use appears to be associated with a significant increase in ASAG detection rate.

### Low overall ASAG prevalence

According to the department registry for the past years, the estimated overall prevalence of ASAG was around 2%, comparable to observed prevalence in our present study (2.53%). However, it remains lower than reported prevalence in the literature concerning the Western population, which ranges between 7% and 45% 3 4 .

One possible explanation is that some studies included patients from centers with dedicated follow-up programs for ASAG, and unlike in our study, these patients were not excluded from the prevalence evaluation 4 .

Another explanation could be the low prevalence of *H. pylori* infection in Belgium, one of the leading risk factors for ASAG 5 14 . In fact, despite including a non-negligible percentage of patients with personal or familial immigration backgrounds, *H. pylori* infection rates remained relatively low in our study population (30.1%), which may have led to reduced ASAG prevalence.

Multiple studies have shown that ASAG more frequently affects the older population 15 16 . Our outpatient average age (46.6 years) was lower than that described in other studies (60 years) 4 . This also could have contributed to lower ASAG prevalence in our population.

Finally, in a prospective Spanish study between 2021 and 2023 that included 998 patients undergoing UGIE, ASAG prevalence was found to be 3.9%, in line with the overall prevalence in our study 17 .

### Association of increased ASAG detection rate with systematic NBI-guided biopsies compared with conventional Sydney protocol with optional NBI use

Despite being statistically non-significant, an increased ASAG detection rate was observed with systematic use of NBI-guided biopsies in phase two ( *P* = 0.269). Various reasons could potentially explain why this result failed to reach statistical significance.

First, this could be explained by the limited sample size of the studied population and subsequent underpowered statistical analysis. Indeed, the initially hypothesized ASAG prevalence of 2% was found to be lower in the first phase of the study (1.43%), indicating the need for a larger sample size (842 patients per phase) to reach adequate statistical power. Given the substantial sample size required for the study, these findings could encourage initiation of a multicenter prospective study, evaluating ASAG prevalence on a larger scale in Belgium.

On another note, the study was designed to compare two attitudes in current practice. Phase 1 of the study represented the most commonly used approach in the real world, particularly by endoscopists with limited NBI experience, whereas phase 2 demonstrated systematic use of NBI for guided biopsies. Consequently, the liberal and ethical concept of phase 1 allowing NBI use at endoscopist discretion may have reduced the disparity between the two phases.

Moreover, given that ASAG primarily affects older individuals, targeting this particular age group, as done in previous studies, may have contributed to a higher reported prevalence of ASAG.

Finally, correct identification of NBI patterns corresponding to ASAG requires a learning curve 18 . In our study, endoscopists had limited experience with use of NBI for detection of gastric precancerous conditions. This may have negatively influenced sensitivity, particularly in the first phase.

In the same way, a numerical increase in the detection rate for atrophy and IM (all stages combined) was observed between phase 1 and phase 2, the overall prevalence of IM and atrophy all stages combined being 17.7% and 16.8%, respectively.

Analysis of the diagnostic performance of endoscopists in both phases showed an increase in sensitivity for detecting atrophy and IM with systematic use of NBI-guided biopsies, statistically significant for IM only ( *P* < 0.001). In contrast, a decrease in specificity was noted, possibly reflecting endoscopist motivation to identify more lesions.

Even when the results of phase 2 failed to show statistical significance favoring systematic use of NBI-guided biopsies, “suspicion-free” NBI use demonstrated a statistical advantage for detection of ASAG ( Table 5 ). These results, supported by other results in the literature 4 6 , convey an important message to our fellow endoscopists, encouraging systematic use of NBI in guiding gastric biopsies, particularly for detection of ASAG.

### *H. pylori* infection and ASAG

*H. pylori* infection prevalence was 30.1% in our population, in line with the low Belgian prevalence reported in the literature (< 40%) 14 . In addition, *H. pylori* infection was more prevalent in the younger population, < 50 years of age (36.1% versus 20.9%) ( *P* = 0.001).

In our study, presence of *H. pylori* was not correlated with presence of ASAG; 27.3% of patients with ASAG were *H. pylori* positive. This differs from the literature where prevalence of CAG was found to be 2.4-fold higher in *H. pylori* -positive patients 19 . This might be explained by unmeasured contributing factors other than *H. pylori* infection, such as bile reflux or high gastric pH 20 . Furthermore, histological changes in the gastric epithelium might be associated with progressive decline in *H. pylori* colonies, which are subsequently replaced by other gastric microbiota 21 . Finally, undisclosed previous *H. pylori* eradication also may have contributed to this finding.

### Risk factors associated with ASAG, IM and atrophy detection

Known risk factors for ASAG include age, sex, BMI, geographical origin, smoking habit, excessive alcohol consumption, family history, and *H. pylori* status 5 . In our population, besides age, none of the previously mentioned factors were identified as being significant. An eight-fold increased risk of acquiring ASAG was observed with age older than 50 years. Risk factors associated with presence of IM and atrophy were age older than 50 years and excessive alcohol consumption. Family history of GC was not a significant risk factor, considering it was solely assessed through patient interviews.

### Limitations

Our study is subject to several limitations. As previously mentioned, randomization of participants was deemed unfeasible in a real-world setting, making it hard, with our non-randomized study design, to demonstrate causality between systematic NBI use with NBI-guided biopsies and increased prevalence of ASAG.

Excluding patients undergoing UGIE under general anesthesia may have influenced prevalence. We hypothesized that suboptimal tolerance during awake endoscopy might preclude visibility 22 . Moreover, UGIE under general anesthesia is often performed concomitant with colonoscopy in patients ≥ 50 years old for screening purposes, which may have increased observed ASAG prevalence.

Belgium is a multilingual country. Informed consent was provided in the three most commonly spoken languages (English, French, and Dutch). However, many patients presenting to Saint-Pierre University Hospital speak other native languages, and therefore, were not eligible to participate. This immigrant population may carry a higher risk of ASAG.

Finally, integration of instructive presentation on NBI use in ASAG detection between the two phases may have affected the results by increasing the incentive of the endoscopists to detect more ASAG cases during phase 2. However, the informative presentation on ASAG prevalence at the beginning of the study and the obligatory consistent use of NBI in phase 2 should have reduced this impact.

## Conclusions

This is the first time an NBI-enhanced Sydney protocol with systematic use of NBI-guided biopsies has been studied in a Belgian population. Observed prevalence of ASAG in our study was consistent with the most recent literature. Systematic use of NBI-targeted biopsies was associated with improved detection rates for ASAG, atrophy, and IM. Furthermore, “suspicion-free” NBI use was found to be associated with significantly increased likelihood of detecting ASAG. Age older than 50 years was the only risk factor associated with ASAG. We hope that these promising results will motivate endoscopists, both at our institution and elsewhere, to prioritize use of NBI-enhanced Sydney protocol with targeted biopsies, particularly in the older population. This is the first step toward increasing ASAG detection and ensuring better follow-up for our patients.
